# Physiological performance of the intertidal Manila clam (*Ruditapes philippinarum*) to long-term daily rhythms of air exposure

**DOI:** 10.1038/srep41648

**Published:** 2017-01-27

**Authors:** Xuwang Yin, Peng Chen, Hai Chen, Wen Jin, Xiwu Yan

**Affiliations:** 1 Liaoning Provincial Key Laboratory for Hydrobiology, College of Fisheries and Life Science, Dalian Ocean University, Dalian, China

## Abstract

Intertidal organisms, especially the sessile species, often experience long-term periodic air exposure during their lives. Learning the biochemical and physiological responses of intertidal organisms to long-term periodic air exposure and the relationship to duration of air exposure provides insight into adaptation to this variably stressful environment. We studied the Manila clam, *Ruditapes philippinarum*, an important species in world aquaculture, as a model to evaluate survival, growth, lipid composition, oxygen consumption, oxidative damage, and antioxidant enzyme activity in relation to the duration of air exposure in a long-term (60 days) laboratory study of varying durations of periodic emersion and re-immersion. Our results show: (1) clams undergoing a longer period of air exposure had lower survival and growth compared to those given a shorter exposure, (2) levels of oxidative damage and activities of antioxidant enzymes were higher in all air exposure treatments, but did not increase with duration of air exposure, and (3) the content of docosahexaenoic acid increased with duration of air exposure. Our results can largely be interpreted in the context of the energy expenditure by the clams caused by aerobic metabolism during the daily cycle of emersion and re-immersion and the roles of docosahexaenoic acid against oxidative stress.

During periods of low tide, intertidal habitants, especially sessile organisms, often face air exposure, resulting in water loss, oxygen deficiency, thermal stress, and limited food supply^1^,^2^,^3^. The adaptations of intertidal organisms to cope with extreme environmental changes during tidal cycles have received many attentions^4^,^5^,^6^,^7^,^8^,^9^,^10^,^11^,^12^. For example, intertidal bivalves evolved two main strategies to deal with air exposure^1^: continuous anaerobic metabolism during valve closure during air exposure, and^2^ intermittent aerobic respiration with periodic gaping^1^,^4^,^10^. The adaptations to anaerobic metabolism provide water conservation, buffering against thermal stress, and protection from predation. However, maintenance of anaerobic metabolism causes rapid depletion of energy reserves and the accumulation of anaerobic end products (e.g., succinate, alanopine, lactate, and ammonia)^1^,^9^,^13^. In contrast, benefits of aerobic respiration include decreased energy expenditure, compensation of oxygen debt, and elimination of anaerobic metabolites but at the cost of evaporative water loss and predation vulnerability^1^,^4^,^14^,^15^.

Some bivalve mollusks (e.g., clams) are permanent dwellers in the intertidal zone. In the field, tide cycles usually subject these organisms to air exposure lasting 0–6 h, depending on the variation in tidal height or shore elevation^1^,^9^,^10^. In response to daily rhythms of emersion and re-immersion, bivalves evolved physiological acclimation or metabolic adaptation to life at different shore levels. For example, low intertidal bivalves close their valves and undergo anaerobic metabolism during emersion, while bivalves inhabiting the high intertidal zone exhibit maintenance of aerobic respiration with gaping behavior^1^,^4^,^10^. Extensive field investigations and empirical studies have been carried out to study the biochemical and physiological responses of intertidal bivalves to short-term (usually from hours to days) air exposure^4^,^10^,^13^,^16^,^17^,^18^. However, there is less information on the acclimation and adaptation of intertidal bivalves to long-term air exposure. In the life history of bivalve mollusks, the existence of planktonic embryos and larvae usually lasts for several weeks. After settling out in the intertidal zone, bivalves like clams spend the rest of their lives in settlement sites, undergoing daily rhythms of emersion and re-immersion for months to years. Evaluating the responses of bivalve mollusks to long-term daily rhythms of air exposure may help in understanding the adaptation of intertidal organisms to environmental heterogeneity.

Field investigations have revealed that both survival and growth of bivalves from high intertidal zones are lower than those of congeners inhabiting lower intertidal zones^11^,^19^. Stressful conditions imposed by air exposure, such as food limitation and respiration restriction, may be more severe in high intertidal zones. For bivalve species without gaping behavior, during tidal withdrawal inhabitants in higher intertidal zones suffer longer periods of anaerobic metabolism than lower intertidal congeners, resulting in the expenditure of energy stores at a higher rate^1^,^20^. When re-submerged, higher intertidal inhabitants also employ higher aerobic metabolism to recover from a larger “oxygen debt”^14^, leading to a more rapid utilization of energy reserves compared to lower intertidal congeners^4^. The joint influence of anaerobic and aerobic metabolism during emersion and re-immersion may explain the variations in the life histories of conspecific bivalves inhabiting different elevations of intertidal zone.

During low tide, intertidal organisms are usually exposed to thermal stress, solar radiation, and hypoxia, which make the production of reactive oxygen species (ROS)^17^,^21^,^22^. Cell respiration produces ROS, the amount of which can be positively correlated with O_2_ utilization^23^. Under stressful conditions, the production of ROS may exceed the capacity of organisms to alleviate them, causing oxidative stress and producing lipid peroxidation^2^,^20^. The elevation of O_2_ respiration in reoxygenation during re-immersion have been suggested to result in the enhanced ROS production in intertidal organisms^3^, whereas the accumulation of succinate in hypoxia may also contribute to ROS production during emersion^20^. To protect them against ROS, a system with enzymatic antioxidants, such as superoxide dismutase (SOD), catalase (CAT), glutathione peroxidase (GPx), and ascorbate peroxidase (APX), was developed by many intertidal organisms^8^,^17^,^22^. Both oxidative damage level and antioxidant ability within a population are sometimes found to be associated with tidal height, suggesting a positive feedback between the duration of air exposure and the antioxidant capability of intertidal organisms^10^,^18^,^24^.To defend against ROS, another system with nonenzymatic antioxidants, e.g., vitamin C, carotenoids, and glutathione (GSH), was also developed in marine organisms^2^. However, the roles of polyunsaturated fatty acids (PUFA), in defense against oxidative stress of marine organisms have received little attention. Lipid peroxidation is usually caused by the reaction of ROS (especially HO^•^) to unsaturated double bonds^25^,^26^. The *n*-3 PUFA, such as eicosapentaenoic acid (EPA) and docosahexaenoic acid (DHA), are more sensitive to the aggression of ROS because of the abundance of multiple double bonds; however, some DHA-enriched organs (e.g., the brain) are ideal targets for ROS, but show weak lipid peroxidation^27^. This paradox can be explained by the hypothesis that EPA or DHA exerts an antioxidant effect by either accelerating the activities of antioxidant enzymes^28^,^29^ or enhancing membrane fluidity^30^,^31^,^32^. In addition, these studies also suggest that DHA is a more potent antioxidant effect promoter than other PUFA^27^,^30^,^31^. The correlation between PUFA and oxidative stress defense is mainly examined in mammals, but it will be interesting to address this question in marine bivalves not only because of the prevalent oxidative stress in marine environments^2^,^3^ but also due to the potential capability of marine bivalves to synthesize n-3 long-chain PUFA (e.g., DHA)^33^.

*Ruditapes philippinarum* (Manila clam) is a common sessile intertidal clam who is usually subjected to cycles of emersion and re-immersion during the tide cycle. When juvenile clams settle in muddy and sandy sediments of the intertidal zone and live buried a few centimeter deep^34^, they experience daily rhythms of air exposure throughout their lives, leading to physiological stresses caused by water loss, oxygen deficiency, thermal stress, and food limitation^1^,^13^,^14^. Both field and laboratory observations reveal the gaping behavior of Manila clams when they are in immersion^15^,^34^,^35^, which is associated with siphon extension behavior and is considered to be related with the feeding^34^,^35^. However, when Manila clams are under hypoxia (e.g. during air exposure), their shell valves are frequently tied up, likely undergoing low oxygen consumption and reduced metabolisms^15^,^16^. In the last decade, the Manila clam has become an important aquaculture species all over the world, and the output of fresh clam reached over 4.0 million tons in 2014^36^. Using the Manila clam as a model organism to evaluate their responses to environmental fluctuation not only adds to our understanding of ecology and evolution of intertidal bivalves, but is also important in improving aquaculture techniques. We evaluate the effects of different air exposure regimens on life history characteristics, physiological performance, and biochemical composition of Manila clam over a 2 month period. The emersion and re-immersion regimens are based on a 24 h daily rhythm. More specifically, we tested the following hypotheses: 1) clams undergoing a longer period of air exposure have lower survival and growth compared to those in shorter exposure regimens, 2) level of oxidative damage and activity of antioxidant enzyme in clams increase with duration of emersion, and 3) the content of DHA in clams increases with the duration of air exposure.

## Results

### Survival and growth of clams

**Figure 1:**
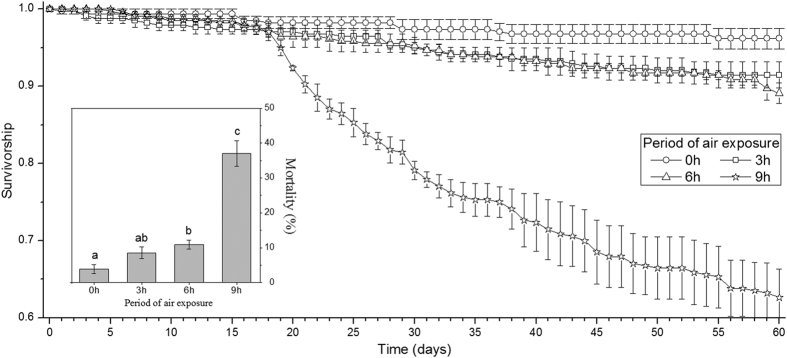
Daily survivorship and total mortality of *Ruditapes philippinarum* in different air exposure treatments (0, 3, 6, and 9 h). Different letters (a,b, and c) in the inserted figure of mortality shows significant differences (*P* < 0.05) among different treatments (0, 3, 6, and 9 h). Data are mean ± standard error based on the four replicated populations. See Fig. 6 for the meaning of 0, 3, 6, and 9 h.

The air exposure duration produced significant differences on the mortality of clam populations (ANOVA, *P* < 0.0001). Clam populations that were exposed to 9 h of daily air exposure had higher mortality rates than other populations (Fig. 1), nearing 40% after 60 days of daily rhythms of emersion. The mortality rate of clams in non-emersion treatment was less than 5% and showed no difference with 3 h of air exposure treatment. During air exposure treatments, no obvious difference was observed in the survival curves of all treatments in the first three weeks (Mann-Whitney *U*-tests, *P* > 0.05, Fig. 1). After the first three weeks, survival of clam populations with 9 h of daily air exposure started showing greater decreases than other populations (Mann-Whitney *U*-tests, *P* < 0.0001, Fig. 1). We also detected greater differences in the survival curves of clam populations between the non-emersion treatment and 3 or 6 h air exposure treatments (Mann-Whitney *U*-tests, *P* < 0.0001) but no difference between the 3 and 6 h air exposure treatments (Mann-Whitney *U*-tests, *P* = 0.971).

**Figure 2:**
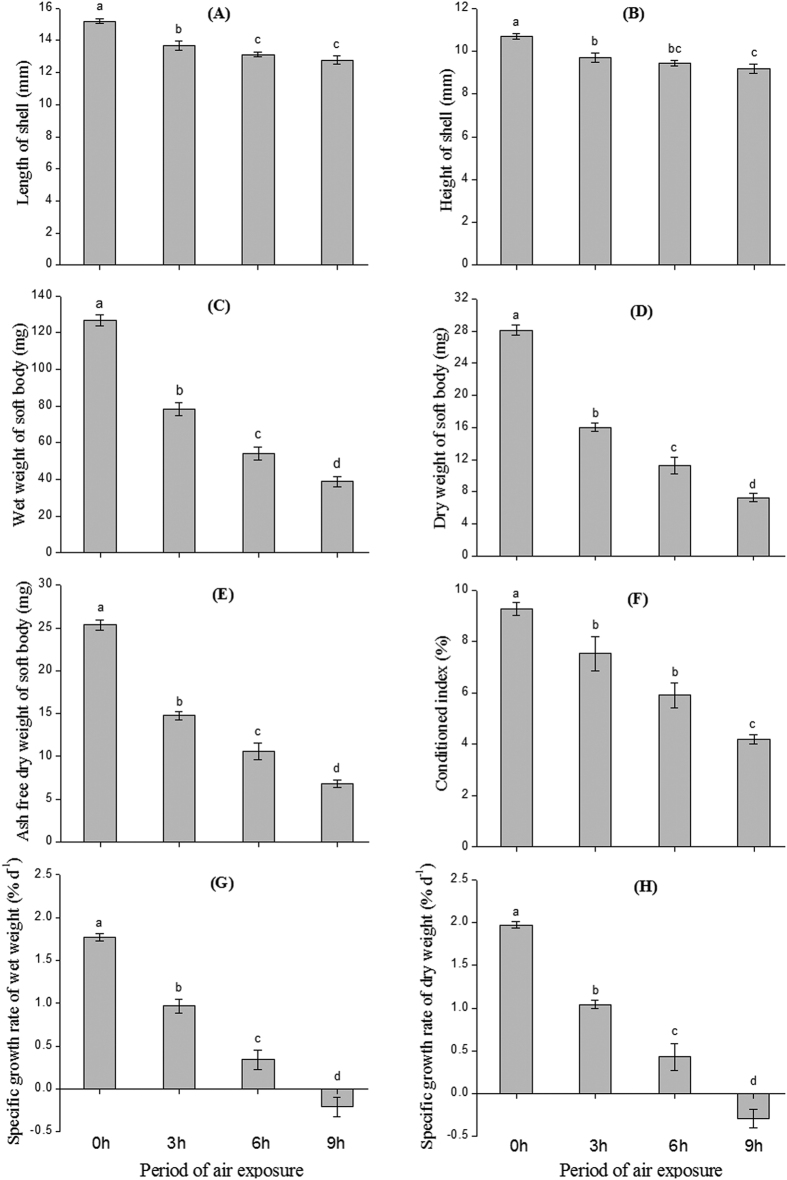
Growth of *Ruditapes philippinarum* after 60 days of laboratory culture with different air exposure regimens. (**A**) Length of shell (mm); (**B**) Height of shell (mm); (**C**) Wet weight of soft body (mg); (**D**) Dry weight of soft body (mg); (**E**) Ash free dry weight of soft body (mg); (**F**) Conditioned index [%, (soft tissue dry weight/shell weight) × 100]; (**G**) SGR of wet weight (% day^−1^); (**H**) SGR of dry weight (% day^−1^). SGR = [100 × (lnW_2_-lnW_1_)]/D, where W_2_ and W_1_ are respectively the final and initial soft body wet (or dry) weight of *R. philippinarum*, and D is the duration of the experiments (60 days). Data are mean ± standard error based on four replicated populations. The value of each replicate is based on the mean of ten individual clams. See Fig. 6 for the meaning of 0, 3, 6, and 9 h.

After 60 days of air exposure treatments, the air exposure duration produced clear differences on all growth parameters of clam populations (ANOVA, *P* < 0.05, Fig. 2). Populations without air exposure showed higher values in length and height of shell, wet and dry weight of soft body, ash free dry weight of soft body, and conditioned index than other air exposure populations (Fig. 2). Clams that were not exposed to air showed the highest positive SGR, whereas clams that were exposed to 9 h air exposure every day showed negative specific growth rate (SGR). Moreover, populations with less air exposure time consistently showed better growth performances than the population subjected to longer air exposure (Fig. 2).

### Oxygen consumption rate (OCR) of clams

**Figure 3:**
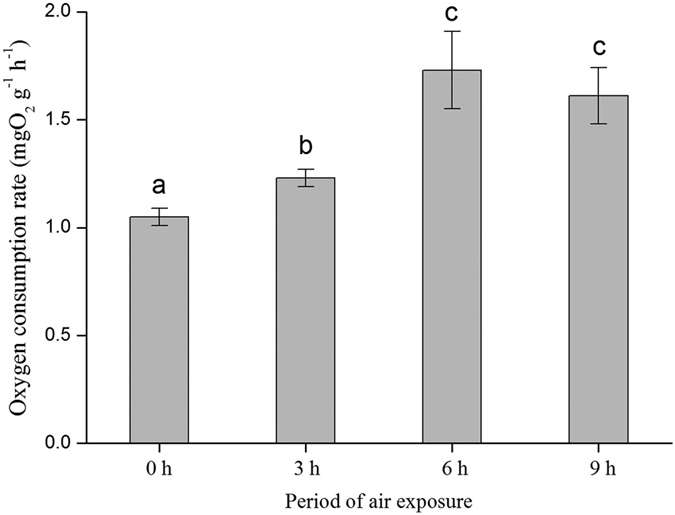
Oxygen consumption rate [mgO_2_ g^−1^ (soft body dry weight) h^−1^] of *Ruditapes philippinarum* in water after long-term culture (60 days) in different air exposure treatments (0, 3, 6, and 9 h). Different letters (a,b, and c) in the figure show significant differences (*P* < 0.05) among different treatments (0, 3, 6, and 9 h). Data are mean ± standard error based on four replicated populations. The value of each replicate is based on ten individual clams. See Fig. 6 for the meaning of 0, 3, 6, and 9 h.

OCR of the clams are shown in Fig. 3, which showed significant differences between the four treatments (ANOVA, *P* = 0.003). Clams in the non-emersion treatment had lower OCR than clams in the other three air exposure treatments, but no OCR difference was detected between the OCR of clams emersed for 6 and 9 h per day.

### Oxidative damage and antioxidant enzyme activity of clams

**Figure 4:**
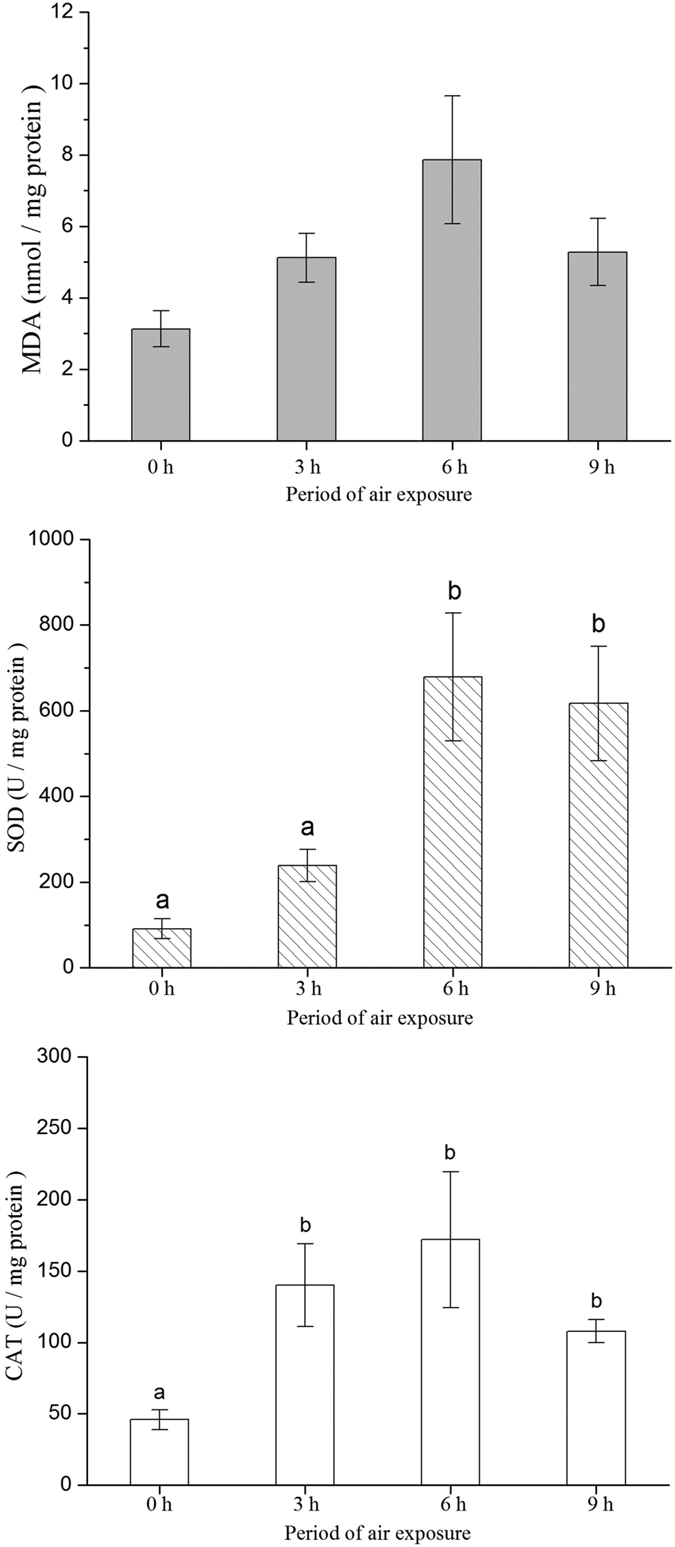
Concentration of oxidative damage to lipids (MDA) and activities of antioxidative enzymes (SOD and CAT) in the whole soft body of *Ruditapes philippinarum* after long-term culture (60 days) in different air exposure treatments (0, 3, 6, and 9 h). Different letters (a, b, and c) in the figure show significant differences (*P* < 0.05) among different treatments (0, 3, 6, and 9 h). Data are mean ± standard error based on four replicated populations. The value of each replicate is based on the mean of three individual clams. See Fig. 6 for the meaning of 0, 3, 6, and 9 h. SOD, superoxide dismutase; CAT, catalase; MDA, malondialdehyde.

The levels of lipid peroxidation showed no significant difference between the four treatments (ANOVA, *P* = 0.066, Fig. 4). Clear differences were observed in SOD and CAT activity of clams under the different treatments (ANOVA, *P*_SOD_ = 0.004, *P*_CAT_ = 0.024, Fig. 4). Clams exposed to air for 6 h consistently demonstrated higher levels of SOD and CAT activity compared with those of clams in other treatments. SOD and CAT activities in clams without air exposure reached relatively low levels, and no difference was found in the CAT activity of clams under 3 and 9 h treatment (Fig. 4).

### Fatty acids composition of clams

**Figure 5:**
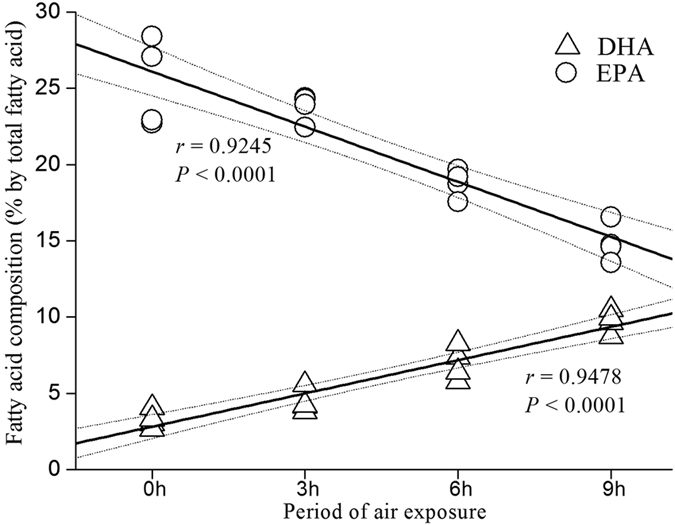
PUFA content (% by total fatty acid) of DHA and EPA in the whole soft body of *Ruditapes philippinarum* in relation to different long-term daily rhythms of air exposure (0, 3, 6, and 9 h). Broken lines indicate 95% confidence limits for the predicted regression lines. Data shown are population means of each replicate. The value of each replicate is based on ten individual clams. DHA, docosahexaenoic acid; EPA, eicosapentaenoic acid. See Fig. 6 for the meaning of 0, 3, 6, and 9 h.

Clam soft body analysis showed no difference in the total lipid content (ANOVA, *P* = 0.920, Table 1). However, changes in the fatty acid composition of clams were observed. The linoleic acid, arachidonic acid, and docosapentaenoic acid content of clams showed no differences between the air exposure treatments (ANOVA, *P* > 0.05), whereas EPA and DHA content revealed great differences (ANOVA, *P* < 0.001). Furthermore, no differences were observed in the amounts of PUFA and saturated fatty acid, but significant differences were observed in the monounsaturated fatty acid content and DHA/EPA ratio (see Table 1). Our data showed that DHA increased linearly, and EPA decreased linearly as the duration of air exposure increased (Fig. 5). Moreover, this finding could be explained by a significant linear regression model (linear regression analysis, *R*_DHA_ = 0.9478, *R*_EPA_ = 0.9245, *P*_DHA_ < 0.0001, *P*_EPA_ < 0.0001).

## Discussion

In this study, we evaluate the responses of life history, biochemistry, and physiology of the Manila clam *R. philippinarum* to long-term (60 days) emersion and re-immersion with 24 h daily rhythm and varied air exposure durations. For the tested clams, survival and growth rates, aerobic metabolism, oxidative damage, antioxidant enzyme activity, and fatty acid composition were all influenced by the air exposure duration. In the field, Manila clam live buried under soft sediments along the mid and low-intertidal zones^34^. Given the buffering capacity of sand or mud cover, clams are usually protected from temperature change and moisture loss during low tides, leaving oxygen deficiency and food limitation as the main stresses during air exposure. Many studies have shown the disadvantageous influences of high temperature, low humidity, and food limitation on the performance of clams during air exposure^15^,^16^,^18^,^19^. In these air exposure treatments, we maintained high relative humidity (87–90%), low air temperature (16–18 °C), and consistent food supply (3 h with 3% of wet weight food). Thus, the responses of clams to air exposure may largely be explained by the physiological stresses due to the limitation of O_2_ respiration.

Our results show that clams undergoing longer air exposure times suffer greater mortality and exhibit lower growth rates than those with short-term or no air exposure. In the 9 h daily air exposure treatment, over 30% mortality rate and negative growth rates of clams were observed during the 60-day air exposure treatments. Our data are in agreement with the field investigations, indicating that growth rate and density of bivalves decline with increasing shore height^11^,^37^,^38^,^39^. Lacking gaping behavior, the Manila clam suffers from anoxia and undergoes anaerobic metabolism during air exposure. Moreover, a positive correlation may exist between the duration of air exposure and levels of hypoxia or anaerobic metabolite accumulation^20^. When clams are re-immersed, they also need to compensate for the oxygen debt caused by hypoxia. In this work, we rule out the effects of feeding time and starvation, which are supposed to be important factors limiting the growth, survival, and energy budget of intertidal bivalves^19^,^37^,^40^. Accordingly, energy expenditure in anaerobic and aerobic metabolism during the daily cycle of emersion and re-immersion, and their positive correlation with air exposure duration can be used in interpreting our findings. However, due to non-gaping behavior in *R. philippinarum* during air exposure, we speculate that our results can largely be explained by the energy expenditure caused by the elevated aerobic metabolisms during re-immersion. Increased oxygen consumption of Manila clam under re-immersion^16^^, present work^ and decreased anaerobic metabolisms of some other clam species under hypoxia^15^,^41^ showed evidences to support our speculation.

When returned to water after air exposure, the aquatic OCR of bivalves increased in comparison with the pre-emersed ones to compensate for the oxygen debt^14^,^16^. Our results verified this finding and showed an increased O_2_ respiration of clams in water after long-term daily rhythm of air exposure compared to the non-emersion samples. However, we did not observe correlations between the duration of air exposure and OCR in the tested clams. Commonly, the size of oxygen debt is proportional to the duration of air exposure period^42^,^43^; but, OCR of clams showed no difference between 6 and 9 h of air exposure treatment. During the middle and late periods of our experiment, inconspicuous gaping behavior (slight opening of the valves) was observed in the tested clams of the 9 h air exposure treatment (personal observation). As mentioned above, aerobic respiration during air exposure can remove the anaerobic metabolites as well as compensate the oxygen debt; thus, physiological stresses experienced by clams may not be as severe under extreme air exposure. Given the observations that *Ruditepes* bivalves can slightly open their valves under extreme hypoxia^15^,^16^, our speculation is possible. The role of gaping in aerobic respiration has been regarded as the adaptation to long periods of air exposure by bivalves inhabiting the upper areas of the intertidal zone, e.g., *Geukensia demissa, Mytilus edulis, Perna perna*, and *Polymesoda expansa*^1^,^4^,^10^,^13^. However, for some bivalves that are widely distributed in the subtidal, intertidal, and upper tidal zones, the responses to air exposure are modified by environmental conditions experienced by bivalves. For example, higher shore clams *Corbicula flunimea* and mussels *M. califormianus* manifest air O_2_ respiration with valve gaping when exposed to air, while their lower shore congeners tend to maintain closed valves^14^,^44^. These differences in terms of response to emersion were also reported in gastropods, e.g., the limpet *Nacella concinna*^9^.

The transitions between hypoxia to normoxia caused by tidal cycles frequently happen in intertidal zone. Thus, mitochondrial ROS generation^45^ during the return of oxygen availability together with the succinate accumulation during hypoxia result in the production of ROS in intertidal mollusks^20^. Some studies have revealed that bivalves usually undergo low metabolic rates under hypoxia^15^,^41^,^46^; hence, enhanced O_2_ respiration of *R. philippinarum* during re-immersion may largely contribute to the ROS accumulation in our work. The contribution of succinate to oxidative stress cannot be ruled out in our work, leaving the hypothesis (e.g., correlation between succinate accumulation and oxidative stress in bivalves during long-term air exposure) to be tested in future studies. Our data showed the increased activities of SOD and CAT of clams under air exposure compared to clams without air exposure. SOD and CAT in marine organisms play important roles in reducing the accumulation of ROS and related deleterious effects, such as DNA damage, protein degradation, and lipid peroxidation^2^. In some cases, levels of ROS are positively correlated with O_2_ respiration magnitude, suggesting the increased activity of antioxidant enzymes in response to ROS production^23^. However, our results do not support this idea, because SOD and CAT activity were not related to the duration of air exposure. Two possible reasons may explain this discrepancy. First, to eliminate the influences of food algae and feces, SOD and CAT activity were measured after 24 h of immersion, to empty the alimentary canal after the experiments. The pathways associated with protein rescue and cellular repair are activated in bivalves as soon as re-immersion occurs^18^. These responses help the organisms in reducing ROS accumulation, decreasing oxidative damage, and recovering from air exposure in a short period^2^,^17^. Re-immersion from air exposure for a whole day may help *R. philippinarum* reduce ROS levels to some extent. Second, other antioxidant systems may contribute in the defense against oxidative stress, e.g., PUFA.

The roles of PUFA in defense against oxidative stress in marine organisms can be deduced from observations that dietary DHA, EPA, and arachidonic acid improve the stress resistance of aquaculture animals^47^,^48^,^49^,^50^,^51^. Given the abundance of multiple double bonds, PUFA may serve as a trap for ROS. In the present work, we did not observe changes in the total PUFA content of clams in relation to the duration of air exposure. However, an interesting pattern found in our data is that DHA is positively related to the period of air exposure, whereas EPA decreases linearly as the duration of air exposure increases. This finding suggests three speculations associated with the Manila clam: 1) the conversion of EPA to DHA caused by air exposure, 2) the role of DHA in defense against oxidative damage, and 3) the positive correlation between DHA content and duration of air exposure. First, the capability of marine bivalves to synthesize n-3 long-chain PUFA (e.g., DHA) has been observed in previous study^33^. Given that contents of total lipid and PUFA were not changed among treatments, DHA is highly likely to be converted from EPA with the synthesis pathway of Sprecher’s shunt (20:5, n-3 → 24:5 n-3 → 24:6 n-3 → 22:6 n-3)^52^. In addition, the synthesis of DHA in clams under hypoxia seems more likely when the findings that DHA can be synthesized in anaerobic and oxygen-limited environments^53^ are considered. Second, possible mechanisms have been suggested for the antioxidant protection effect of DHA. One is that membrane raft function of phospholipids and membrane fluidity may be improved with the supplement of DHA but not EPA^30^,^31^,^32^,^54^. Another mechanism is the possible capability of DHA to accelerate the antioxidant capacity of the existing enzymatic and nonenzymatic antioxidants and to reduce the accumulation of ROS^27^,^55^. Third, the reactive pathway of ROS diversion after DHA treatment suggests a stoichiometric relationship between the amount of DHA and the reduction of ROS^55^. Frequently, the accumulation of ROS is supposed to be positively related to both the period and the magnitude of stress conditions. Therefore, we can expect a positive feedback between the duration of air exposure and the DHA content of Manila clams. The speculations mentioned above can also explain the similar MDA concentrations of clams under different treatments. Given that levels of MDA have been measured as indicator of lipid peroxidation in marine organisms^17^,^56^, the weak lipid peroxidation of clams under hypoxia may be interpreted as the results of high DHA content.

In conclusion, the intertidal *R. philippinarum* exposed to long-term daily rhythms of air exposure showed diminished growth and survival when compared to the groups without air exposure. For the non-gaping *R. philippinarum*, we speculate that this finding may be largely due to the energy expenditure caused by the elevated aerobic metabolisms during re-immersion. After long-term air exposure, the DHA of *R. philippinarum* increased linearly with prolonged duration of air exposure, presumably having been converted from EPA. The enhanced DHA content may account for the oxidative stress induced by air exposure. Given the roles of DHA in defense against ROS, we observed the weak lipid peroxidation in *R. philippinarum* under hypoxia. Certainly, it should be pointed out that our data may underestimate the influence of long-term air exposure on physiological performance of *R. philippinarum* in natural conditions. For example, during tidal cycles, physiological stress of *R. philippinarum* from higher intertidal zones will be more severe because of the temperature fluctuation and feeding deficiency accompanied with air exposure. Given that intertidal organisms frequently encounter multiple stresses during tidal cycles, the interactions of long-term air exposure and a variety of environmental stresses (e.g., temperature, salinity, and pollution) leave issues open for future studies. Furthermore, our work may be helpful in developing improvements in the aquaculture of the Manila clam with different commercial purposes: 1) shortening the production period of clams by rearing them in sub-tidal or lower intertidal zones, and 2) enhancing nutrient values (e.g., DHA) by culturing organisms in higher intertidal zones.

## Methods

### Clam cultures and air exposure regimens

Spats of the Manila clam were collected at 12 °C from the aquatic seed breeding farm of Tianjin Aquaculture University, P.R. China in March 2014. We used spats in the present work because juvenile clams are usually incubated indoors until they become spats of approximately 10 mm shell length, and then they are reared in sand or mud flats along the intertidal zone until they reach market size^57^. In the laboratory, the clams were cultured in glass aquaria (800 × 450 × 450 mm^3^), and the temperature of rearing seawater (32–34 ppt, filtered with a sand filter tank) was gradually increased by 1 °C per day until it reached 18 °C and was kept at 18 ± 1 °C for a week for acclimation. The water temperature was controlled by a temperature regulation system (HXSWT-301, Huixin Titanium Equipment Development Co. Ltd., Dalian, China) with a precision of ± 1 °C, and the rearing seawater was renewed daily. The incubation was conducted with continuous aeration and dim illumination (covering windows with curtains). During the experiment, the temperature of fresh seawater from sand filter tank was 10–12 °C. To minimize the potential physiological stress caused by temperature fluctuations^6^,^7^,^58^, the fresh seawater was heated to 18 °C before use. During the experiment, clams were fed daily with commercial condensed *Nannochloropsis* sp. (density ≈2 × 10^10^ cells g^−1^, Panjin Crab Industry Co., Ltd., Panjin, China) at a density of 3% of the wet weight of the clams. The alga *Nannochloropsis* sp. contains relatively high levels of EPA (38.39%) but low levels of DHA (0.56%)^59^. Although the survival and growth of clams are better in sand or mud, we did not include these substrates in the experiments because: 1) the technique of incubating clams without sediment has been well developed and used in many studies^15^,^34^, and 2) sand or mud could clog temperature regulation systems and impair temperature control.

**Figure 6:**
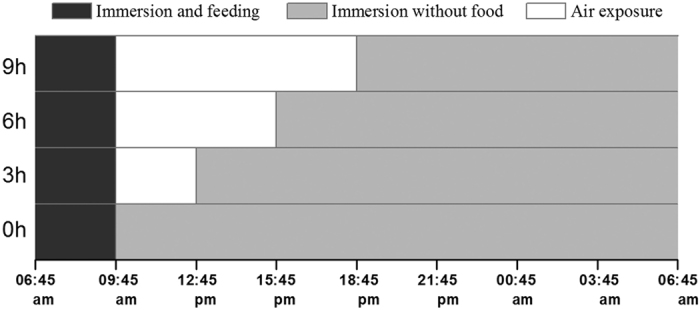
Daily air exposure of different treatments (0, 3, 6, and 9 h) of *Ruditapes philippinarum*. *R. philippinarum* in all treatments were fed with *Nannochloropsis* sp. for three hours every day (from 06:45 am to 09:45 am, black grey bars). After feeding, all the seawater in glass aquaria were refreshed with sand filter seawater and *R. philippinarum* in treatments 3, 6, and 9 h started air exposure after 3, 6, and 9 h, respectively (white bars). When air exposure ended, the *R. philippinarum* in different treatments (3, 6, and 9 h) were re-submerged in renewed seawater (light grey bars). The *R. philippinarum* in 0 h treatment were constantly immersed in seawater. The emersion and re-immersion regimens in different treatments were based on 24 h daily rhythm. After 60 days (from 6:45 a.m. of April 4^th^ 2014 to 6:45 a.m. of June 3^rd^ 2014) air exposure with daily rhythms, as described above, the experiments were terminated. Four replicated experimental populations of *R. philippinarum* were included in each treatment.

After acclimation in the laboratory for two weeks, the clams were randomly allocated to four treatments (0, 3, 6, and 9 h). Clams in treatment 0 h were kept immersed in seawater throughout the experiments, which simulated clams residing in the subtidal zone. In the intertidal zone, inhabitants are usually exposed to air for less than 6 h^9^,^10^,^12^, while those in the high intertidal may undergo longer periods of air exposure^5^. For the experiment, clams in treatments 3, 6, and 9 h were respectively subjected to continuous air exposure for 3, 6, and 9 h every day, to simulate the situations of clams at various intertidal zones. The emersion and re-immersion regimes in different treatments were based on a 24 h daily rhythm (Fig. 6). It is worth noting that the laboratory is located near a coaster zone (38°51′ N, 121°32′ E) in Dalian, China, which is mainly semidiurnal tide sea. Thus, varied durations of air exposure designed in the present work match the natural circumstances to some extent. Clams in all treatments were fed with *Nannochloropsis* sp. for 3 h daily, which allowed the satiation of the clams. In each treatment, the population consisted of 85 clams per glass aquaria, which was similar to the stocking density in the field^36^. For the periods of air exposure, clams were collected and placed in plastic trays (260 × 180 × 60 mm^3^) and were left on the table in the laboratory. To simulate the moist conditions of the intertidal, clams undergoing air-exposure were showered with seawater every 3 h. During the experiment, the room temperature was 16–18 °C, and relative humidity was 87–90%. Each treatment contained four replicated populations, totaling 16 experimental populations in this study. The air exposure treatments began at 6:45 a.m. on April 4, 2014 and ended at 6:45 a.m. on June 3, 2014, lasting for 60 days.

### Survival and growth

Before the air exposure treatments, 40 clams were measured after 24 h starvation to obtain an initial weight and size of clams (length of shell = 13.0 ± 0.3 mm; height of shell = 9.3 ± 0.2 mm; wet weight of soft body = 43.7 ± 6.3 mg; dry wet of soft body = 8.6 ± 0.8 mg; n = 40). The size of clams was measured with Vernier calipers to the nearest 0.02 mm, and the soft body wet weight of clams was measured with an electronic balance to the nearest 0.1 mg. After measuring the wet weight, the soft body was dried at 60 °C for 24 h, then weighted to determine a dry weight to the nearest 0.1 mg.

During the air exposure treatments, the number and occurrence of dead clams were recorded daily, and these were immediately removed from the aquaria. The dead clams were characterized by the marked shrinkage of mantle and large opening of valves. When the air exposure treatments ended after 60 days, 10 clams from each of the 16 populations were randomly sampled, with final weight and size determined individually with the methods described above. Ash content of the soft body was obtained by combustion at 450 °C for 8 h. Growth in each population was based on the mean weight of the ten individual clams.

We also calculated the specific growth rate (SGR) in terms of the wet and dry body weight with the following formula: SGR (%, day^−1^) = 100(lnW_2_-lnW_1_)/D, where W_2_ and W_1_ are respectively the final and initial soft body weight of clams, and D is the duration of the experiment (60 days).

### Oxygen consumption rate (OCR)

In intertidal bivalves, two kinds of OCR are usually evaluated: O_2_ respiration in air and in water. However, for non-gaping Manila clam, whose shells are closed during air exposure, O_2_ respiration in air is weak and cannot be detected^16^. Therefore, we only measured the OCR of clams in water in this work.

After air exposure treatments, five clams from each of the 16 populations were randomly collected and starved for 24 h to minimize the associated metabolic responses. Prior to the determination of oxygen consumption, the clams were gently cleaned of surface detritus, then placed into a 1 L conical iodine flask filled with fresh seawater (0.45 μm fibers filtered) and incubated at 18 ± 1 °C for 24 h. For each population, two replicates and one blank control were used to correct for the respiration of bacteria and organic matter in the seawater. Oxygen content was determined with the Winkler method, and OCR per hour was calculated with the following formula: OCR (mgO_2_ g^−1^ h^−1^) = (D_t_V_t_-D_0_V_0_)/WT, where D_t_ is the change in the oxygen content (mgO_2_ L^−1^) before and after incubation in the tested flasks; D_0_ is the change in oxygen content (mgO_2_ L^−1^) before and after incubation in the control flasks; V_t_ is the volume of the tested flask (L); V_0_ is the volume of the control flask (L); W is the soft body dry weight of the tested clams (g); T is time duration.

### Oxidative damage and antioxidant enzyme

After the experimental treatments, three clams from each of the 16 populations were randomly chosen and starved for 24 h. The soft body of each clam was homogenized in a glass homogenizer for 3 min with 10 mL g^−1^ of ice-cold 0.05 PBS buffer (pH 7.4). The homogenate was centrifuged at 3000 × g for 10 min at 4 °C. The supernatant was kept at −80 °C to measure the level of oxidative damage and activity of antioxidant enzymes. The value of oxidative damage and antioxidant enzyme activity for each population was based on the mean of the three replicates. In this study, we used the concentration of malondialdehyde (MDA), the secondary product of lipid peroxidation, as the measure of oxidative damage. We also measured the activity of the antioxidant enzymes, SOD and CAT, to show the antioxidant capability of the clams.

The concentration of MDA and activity of SOD and CAT were determined according to the manual of the analyses kits used (Nanjing Jiancheng Bioengineering Institute, China). The levels of lipid peroxidation were evaluated according to the MDA product with 2-thiobarbituric at 532 nm^60^. SOD (EC 1.15.1.1) activity was measured by a method defined as the amount of SOD that inhibits the reduction of nitroblue tetrazolium by 50%^61^. CAT (EC 1.11.1.6) activity was measured according to the reactive product of ammonium molybdate with residual H_2_O_2_ at 405 nm^62^.

### Fatty acids composition

After the air exposure treatments, ten clams from each of the 16 populations were randomly collected and starved for 24 h. The lipid content and fatty acid compositions were determined following the methods in previous study^59^. In brief, the fresh soft bodies of the 10 clams were mixed and ground with dichloromethane:methanol. This solution was mixed with potassium chloride and then centrifuged. The lipids were obtained from the lower organic layer, and the solvent was evaporated under nitrogen flow. Total lipids were determined gravimetrically after evaporating the solvent from 5 mL of the purified extraction. Then, 100 mg of lipids were used for lipid class determination by gas chromatography. The identifications of fatty acids were based on comparisons of retention times with known fatty acid methyl ester standard mixtures (37-component FAME mix and menhaden oil, Sigma, Chemical Co, USA).

### Data analysis

Non-parametric Mann-Whitney *U*-tests were used to compare the survival curves of clam populations under different air exposure regimens. Other data sets were analyzed by one-way ANOVA with air exposure duration as the factor after Levene’s test for homogeneity of variances and Kolmogorov-Smirnov test for normality. When significant differences (*P* < 0.05) were detected, Duncan’s test was used for pairwise comparisons. All statistical analyses were carried out using the SPSS statistical package version 21.0. Least square linear regression analysis was conducted to determine the relationship between the air exposure duration and the EPA or DHA content in clams using OriginPro version 8.0. The data, expressed as percentage, were arcsine transformed.

## Additional Information

**How to cite this article**: Yin, X. *et al*. Physiological performance of the intertidal Manila clam (*Ruditapes philippinarum*) to long-term daily rhythms of air exposure. *Sci. Rep.* **7**, 41648; doi: 10.1038/srep41648 (2017).

**Publisher's note:** Springer Nature remains neutral with regard to jurisdictional claims in published maps and institutional affiliations.

## Figures and Tables

**Table 1:** Lipid (% by dry weight) and fatty acid composition (% by total fatty acid) of *Ruditapes philippinarum* in different air exposure regimes.

	0 h – daily air exposure	3 h – daily air exposure	6 h – daily air exposure	9 h – daily air exposure	*P*-value of ANOVA
**Lipid**	5.62 ± 1.06	6.25 ± 0.66	6.03 ± 0.52	6.23 ± 0.47	0.920
**Fatty acid composition**					
C 14:0	1.46 ± 0.16	1.21 ± 0.09	1.32 ± 0.14	—	0.445
C 16:0	21.05 ± 0.57^b^	21.46 ± 0.62^b^	20.17 ± 0.55^b^	17.37 ± 1.03^a^	0.004
C 16:1 n-7	2.63 ± 0.29	2.63 ± 0.15	2.96 ± 0.28	3.18 ± 0.27	0.345
C 16:1 n-9	6.50 ± 0.51^b^	6.39 ± 0.28^b^	5.77 ± 0.49^b^	4.23 ± 0.42^a^	0.011
C 17:0	0.89 ± 0.03^a^	1.02 ± 0.03^b^	1.15 ± 0.02^b^	1.33 ± 0.06^c^	<0.001
C 17:1 n-7	1.68 ± 0.17	1.37 ± 0.13	1.42 ± 0.12	1.59 ± 0.13	0.367
C 18:0	6.85 ± 0.33^a^	7.30 ± 0.26^ab^	7.97 ± 0.18^bc^	8.74 ± 0.27^c^	0.002
C 18:1 n-7	3.71 ± 0.17	3.71 ± 0.10	3.28 ± 0.20	2.99 ± 0.29	0.066
C 18:1 n-9	2.58 ± 0.06^a^	2.65 ± 0.05^ab^	3.13 ± 0.07^bc^	3.84 ± 0.32^c^	0.001
C 18:2 n-6 (LOA)	1.26 ± 0.08	1.22 ± 0.03	1.29 ± 0.04	1.27 ± 0.05	0.758
C 20:1 n-9	4.85 ± 0.29^a^	5.00 ± 0.07^a^	6.12 ± 0.18^b^	7.04 ± 0.04^c^	<0.001
C 20:2 n-6	1.06 ± 0.10^a^	1.22 ± 0.21^a^	1.87 ± 0.12^a^	3.49 ± 0.46^b^	<0.001
C 20:3 n-6	1.08 ± 0.02	1.00 ± 0.06	—	—	—
C 20:3 n-9	1.48 ± 0.07	1.54 ± 0.04	1.73 ± 0.11	1.66 ± 0.04	0.109
C 20:4 n-6 (ARA)	6.91 ± 0.25	6.54 ± 0.18	6.61 ± 0.05	6.56 ± 0.37	0.670
C 20:5 n-3 (EPA)	25.28 ± 1.45^c^	23.77 ± 0.45^c^	18.81 ± 0.45^b^	14.89 ± 0.62^a^	< 0.001
C 22:1 n-7	—	—	—	1.30 ± 0.05	—
C 22:2 n-6	2.44 ± 0.31^a^	2.72 ± 0.24^a^	3.80 ± 0.34^b^	4.68 ± 0.14^c^	<0.001
C 22:4 n-6	2.65 ± 0.24	2.52 ± 0.17	2.83 ± 0.16	3.18 ± 0.23	0.163
C 22:5 n-3 (DPA)	2.61 ± 0.14	2.53 ± 0.10	2.59 ± 0.21	2.97 ± 0.09	0.185
C 22:6 n-3 (DHA)	3.26 ± 0.30^a^	4.45 ± 0.38^a^	6.96 ± 0.55^b^	9.70 ± 0.37^c^	<0.001
**ΣSFA**	31.09 ± 0.51	32.22 ± 0.67	32.48 ± 0.45	30.93 ± 0.33	0.119
**ΣMUFA**	21.94 ± 0.47^a^	21.75 ± 0.18^a^	22.67 ± 0.35^a^	24.16 ± 0.33^b^	0.001
**ΣPUFA**	48.04 ± 0.91	47.25 ± 0.79	47.72 ± 0.79	48.40 ± 0.76	0.481
**DHA/EPA**	0.13 ± 0.02^a^	0.19 ± 0.02^a^	0.37 ± 0.04^b^	0.66 ± 0.05^c^	<0.001

Data are mean ± standard error based on four replicated populations. The value of each replicate is based on 10 individual clams. LOA = linoleic acid; ARA = arachidonic acid; EPA = eicosapentaenoic acid; DHA = docosahexaenoic acid; DPA = docosapentaenoic acid. SFA = saturated fatty acid; MUFA = monounsaturated fatty acids; PUFA = polyunsaturated fatty acids. Different letters (a, b and c) shows significant differences (*P* < 0.05) between different treatments for each fatty acid. See the Fig. 6 for the meaning of 0 h, 3 h, 6 h and 9 h.
